# Evaluation of Acute Leukaemias by Flow Cytometry and Its Correlation With Diagnosis Using Morphological and Special Staining Techniques

**DOI:** 10.7759/cureus.54126

**Published:** 2024-02-13

**Authors:** Nishat Ahmad, Nisha Kumari, Deepali Tirkey, Sunil K Mahto, Moazzam Jawaid

**Affiliations:** 1 Pathology, Rajendra Institute of Medical Sciences, Ranchi, IND; 2 Oral Medicine and Radiology, Sarjug Dental College and Hospital, Darbhanga, IND

**Keywords:** mao, pas, cytochemical stains, flow cytometry, acute leukaemia

## Abstract

Background: Leukaemia can be reliably diagnosed and classified by the simultaneous application of multiple techniques. Cytochemical stains that are cheap and do not require any special instruments are very important in developing countries for the diagnosis of acute leukaemia (AL).

Aim: To diagnose AL in all suspected cases by flow cytometry and to correlate the diagnosis with morphological and special staining like myeloperoxidase (MPO) and periodic acid-Sciff (PAS) techniques.

Methods and materials: The study participants' peripheral blood smear details and bone marrow aspirate smear morphologic findings, as well as socio-demographic information, were taken from the patients' medical files. In total, 57 newly diagnosed instances of acute leukaemia confirmed by flow cytometry were incorporated into the study, which underwent cytochemical labeling and morphological diagnosis. All patients who gave previous consent had their bone marrow aspirated, and a Wright-stained smear was produced for microscopic inspection, cytochemical staining, and immunophenotyping. In an ethylenediaminetetraacetic acid (EDTA) container, peripheral blood was also drawn for the same purpose. During the entire bone marrow smear examination, we used both MPO and PAS staining techniques.

Results: The study was carried out between July 2019 and June 2020. Out of 57 cases in the study, 29 (50.9%) cases on cytochemical analysis of leukaemia using PAS and MPO were diagnosed with acute myeloid leukaemia (AML) and 28 (49.1%) were diagnosed as acute lymphoid leukaemia (ALL). Cytochemical analysis of leukaemia using PAS and MPO rendered the diagnosis in 92.9% of acute leukaemia cases in our study. A total of 25 out of 25 AML cases and 28 out of 32 cases of ALL were correctly diagnosed based on morphology and cytochemical staining. Morphology and cytochemical analysis alone were unable to correctly diagnose a total of four ALL cases. All AML cases that were wrongly diagnosed as ALL were mostly M0 and M1-AML.

Conclusion: Morphological staining diagnosis by itself is capable of correctly identifying a large proportion of cases of AL, which comprised 92.98% of total cases. There was also a favorable relationship between findings of diagnosis by flow cytometry and findings of diagnosis by morphology assessment in determining acute leukaemias.

## Introduction

Leukaemias are diseases in which abnormal proliferation of hemopoietic cells causes progressively increasing infiltration of the bone marrow or, in certain forms, the lymphatic tissues. It can also be described as a malignant clonal illness that, if not treated promptly, progresses quickly to death because it involves the amplification of premature, ill-differentiated blast cells within the bone marrow [1,2]. Lymphoid and non-lymphoid leukaemias are two major variants of leukaemia according to the cell of origin, signs and symptoms, course of disease, and outcome after medical intervention. On the grounds of the disease's progression, the expected outcome, and other factors, they are more frequently separated into chronic as well as acute varieties [3,4].

By using several techniques concurrently, leukaemia can be accurately identified and categorized. In addition to multiparameter flow cytometry, these also comprise cytochemistry, histomorphology, and cytomorphology, which designate the diagnostic sample to the appropriate entity [5,6]. For the diagnosis to be certain, additional chromosomal examinations are also required [7,8]. Because they are inexpensive and don't require any specialized equipment, cytochemical stains are crucial for the identification of acute leukaemia (AL) in underdeveloped nations. It gives hints about the kind and course of cellular differentiation, although it should still be viewed as complementary to morphological analysis rather than a replacement for it [9,10]. Whenever leukaemia is not structurally obvious with Romanowsky stains, cytochemical stains help examine the structure of cells that are still developing.

A lysosomal enzyme called myeloperoxidase (MPO) is found in the azurophilic granules of neutrophils and their precursor cells, eosinophils, and monocytes. The major purpose of its' staining is to distinguish between acute lymphoblastic leukemia (ALL) and acute myeloid leukemia (AML). Periodic acid-Schiff (PAS) is a staining method used to detect polysaccharides, manifested as glycogen, glycoproteins, glycolipids, and mucin in tissues. In general, the PAS stain is recommended to be performed on tissues fixed with 10% neutral buffered formalin. Many autoantibodies and complements are indeed glycoproteins, MPO and PAS are important cytochemical stains [11,12].

Because each subtype of leukaemia has a different therapy option and future outcomes, flow cytometry's ability to differentiate is crucial. The significance of flow cytometry in the detection and categorization of leukaemia has increased as a result of several developments in the field of multiparameter computational approaches [13,14]. The importance of flow cytometry in leukaemia cell profiling is to identify the lineage of cells (B lymphoid, T lymphoid, and myeloid) and to identify the two-pronged origin in biphenotypic leukaemia, unexpected simultaneous expression of antigens, or anomalous profile, and to indicate clonality [15-18].

There have been very few studies that have evaluated the reliability of results from cytochemical stains like MPO and PAS in the diagnosis of AL compared with results from flow cytometry. For this purpose, the diagnosis of AL with flow cytometry can be considered standard criteria. Therefore, this study was carried out to diagnose acute leukaemia in all suspected cases by flow cytometry and correlate the diagnosis with morphological and special staining (MPO and PAS) techniques. These observations may prove to be a useful basis for comparison at a future date.

## Materials and methods

Between July 2019 and June 2020, individuals with AL who had been evaluated at the Pathology Department's Haematology division at Ranchi's Rajendra Institute of Medical Sciences (RIMS) participated in the study. Hematological anomalies like leukemia are detected and treated at RIMS, a specialized referral hospital in the Jharkhand state capital. This serves as the focus of the department's Haematology Section. The common diagnostic techniques employed were cytochemical staining, bone marrow aspiration, and morphological evaluation of peripheral blood. Using flow cytometric analysis on bone marrow specimens and peripheral blood smears, the immunophenotyping of acute leukemia has been carried out. 

The source population consisted of both adult and pediatric patients who sought treatment at various RIMS outpatient clinics for a variety of symptoms and who needed bone marrow aspiration and morphological analysis of peripheral blood at the Haematology Section of RIMS Ranchi. Acute leukemia patients who were admitted to the hospital made up the study's subject population. 

Patients meeting the following specific criteria were eligible for inclusion in this research study. Individuals with acute leukemia who exhibited more than 20% of blasts in their bone marrow and peripheral blood samples through morphological analysis, patients of all ages, and only individuals who had voluntarily agreed to be part of the study were included. Following exclusion criteria were established to delineate specific patient groups not suitable for inclusion in the study. Individuals with a leucoerythroblastic reaction were also excluded. Lastly, patients who were unwilling to participate in the research were excluded from the study.

The study participants' peripheral blood smear details and bone marrow aspirate smear morphologic findings, as well as socio-demographic information, were taken from the patient's medical file. In total, 57 newly diagnosed instances of acute leukaemia confirmed by flow cytometry were incorporated into the study, which underwent cytochemical labeling and morphological diagnosis. Every patient who had provided prior consent underwent a bone marrow aspiration procedure. As part of the assessment, a smear was created using Wright stain for microscopic examination, and further evaluation involved cytochemical staining and immunophenotyping. In an ethylenediaminetetraacetic acid (EDTA) container, peripheral blood was also drawn for the same purpose. Throughout the whole bone marrow smear, cytochemical staining was carried out using both MPO and PAS. To achieve consistent and accurate cell counting, we followed a standardized procedure. We began by using a blood sample measuring 100 microliters (µl). To calculate the cell count, we counted the cells present in this specific volume. Once the cell count in the 100 µl sample was determined, we adjusted it to a concentration of 10,000 cells per microliter (µl). This adjustment involved multiplying or diluting the sample as needed to reach the desired cell concentration of 10,000 cells per µl for further analysis and interpretation. In cases where the cell count was low, gradient separation was performed to concentrate cells, whereas, in instances of high cell counts, samples were appropriately diluted with sheath fluid.

Procedure for myeloperoxidase staining

For myeloperoxidase staining, we utilize two solutions. Solution A comprises 0.3 gm of benzidine dissolved in 99 ml of ethyl alcohol, with the addition of 0.1 ml of a supersaturated solution of sodium nitroprusside. In contrast, Solution B is freshly prepared before use and consists of six to eight drops of hydrogen peroxide in 25 ml of distilled water. This staining method is employed on air-dried peripheral smears and bone marrow films. Ten drops of Solution A are added to the slides and allowed to stand for 1.5 minutes, followed by the direct addition of Solution B (double the volume of Solution A) for a 4.5-minute incubation. After thorough washing with running tap water for 1.5 minutes, the slides are air-dried and counterstained with Leishmann stain to differentiate myeloid series from lymphoid and erythroid series, and further categorize myeloblasts into type I, II, and III based on granular content.

Procedure for periodic acid-Schiff reaction

For the PAS reaction, a formalin-alcohol mixture (1:9) is used as a fixative. The 1% periodic acid, dissolved in 1000 ml distilled water, serves as the primary reagent, along with Schiff's reagent prepared as per De Tomasi Coleman’s method. This involves dissolving 1 gm of basic fuchsin in 200 ml of boiling distilled water, followed by cooling to 50°C and adding 2 gm of sodium metasulphate. After mixing, 2 ml of concentrated HCl is added, and the solution is kept in the dark for 24 hours. Following the addition of activated charcoal, the solution is filtered using Whatman’s filter paper. Nuclei appear blue, while PAS-positive material appears pink to bright red.

Procedure of immunophenotyping by flow cytometry

In the context of suspected ALs, immunophenotyping by flow cytometry involves specific procedural steps. Five labeled falcon tubes, marked one to five, are utilized. Each tube receives 100 µl of count-adjusted blood. Fluorescent dye-conjugated monoclonal antibodies are added to each tube, except the first tube, which serves as a blank. After a 15-minute incubation in the dark at room temperature, 2 ml of lysing solution (diluted 1:10 by distilled water) is added to each tube. Subsequently, the contents are mixed and incubated for 10-15 minutes. The supernatant is discarded, the pellet is broken, and 0.5 ml of sheath fluid is added for analysis in a pre-calibrated flow cytometer. Each tube is designed to assess specific markers, such as CD45, CD20, CD10, CD38, CD19, CD34, CD8, CD5, CD3, CD4, CD7, CD64, CD33, HLA-DR, CD13, CD117, CD79a, and cytoplasmic MPO, tailored to evaluate various cell types and abnormalities in suspected acute leukemia cases.

Interpretation

All the bone marrow or peripheral blood samples were analysed by FACS Canto II flow cytometer (six-color, two-laser; BD Biosciences, San Jose, CA, USA). The results obtained from the flow cytometry analysis were displayed and analyzed using computer software. Dual-parameter dot plots were employed, featuring two parameters on the X and Y axes. To facilitate visualization of the population of interest, gating was employed. Specifically, for the assessment of acute leukemias, a CD45/side scatter gate was utilized. In cases of suspected lymphomas, gating involved the use of both CD45/side scatter and CD19 gating. These gating strategies, aided in distinguishing and characterizing specific cell populations relevant to the respective hematological conditions under investigation.

At least 30,000 total cells were acquired, and the side scatter versus CD45 PerCP dot plot was used for blasts gating. The percentage of positive cells more than 20% was considered positive for that surface or intracellular markers.

## Results

Out of 57 cases in the study, 29 were AML (50.9%) and 28 were ALL (49.1%) which was approximately 1:1. Among the 28 cases of ALL, 12 cases were of T-cell ALL and 16 cases were of B-cell ALL. Table 1 depicts the distribution of AML cases and ALL cases according to age and gender. 

**Table 1:** Age-group-wise distribution and gender details of acute myeloid leukaemia (AML) and acute lymphoid leukaemia (ALL).

Variables	AML (29)		ALL (28)		Total (57)	
	No.	%	No.	%	No.	%
Age Groups (Yrs.)						
0-10	0	0.0%	17	60.7%	17	29.8%
11- 20	2	6.9%	9	32.1%	11	19.3%
21-30	6	20.7%	1	3.5%	7	12.3%
31-40	4	13.8%	0	0%	4	7.0%
41-50	8	27.6%	0	0%	8	14.0%
51-60	5	17.2%	0	0%	5	8.8%
61-70	3	10.3%	0	0%	3	5.3%
>71	1	3.4%	1	3.6%	2	3.5%
Total	29	100%	28	100%	57	100.00%
Gender						
Male	15	26.31%	20	35.08%	35	61.39%
Female	14	24.56%	8	14.02%	22	38.59%
Total	29	50.86%	28	49.11%	57	100%

This chart shows that ALL has a greater predilection for lower age groups, with the maximum number of cases in the age group 0-10 years (n = 17, 60.7%), whereas nine cases (32%) were found to be in the age group of 11-20 years. In AML, cases were clustered in the age group 21-60 years (n = 23, 79.3%). Six cases (20.6%) were in the 21-30 age group. Four cases (13.8%) were in the 31-40 age group. Eight cases were in the 41-50 age group, which was 25.6% of AML cases. The 51-60-year-old age group had five cases (17.2%) (Table 1). Table 2 shows the correlation of morphologic diagnosis versus flow diagnosis in 57 cases.

**Table 2:** Correlation of morphologic diagnosis versus flow diagnosis in 57 cases. AML: acute myeloid leukaemia; ALL: acute lymphoid leukaemia

Morphological Diagnosis	Cases	Flow Diagnosis	TOTAL
AML	25	AML	25 (100%)
		ALL	0 (0%)
ALL	32	ALL	28 (87.5%)
		AML	4 (12.5%)
Total	57	-	57

The overall incidence ratio of M to F was 1.6:1. For AML, the ratio of M to F was 1.07:1. Similarly, the gender ratio in ALL was 2.5:1. So, in our study, there was an overall male preponderance in both AML and ALL, with ALL showing a slightly higher male preponderance as compared to AML.

A total of 25 out of 25 AML cases and 28 out of 32 cases of ALL were correctly diagnosed based on morphology and cytochemical staining. The M3-AML (acute promyelocytic leukemia) was the most common. Morphology and cytochemical analysis alone were unable to correctly diagnose a total of four ALL cases. The AML cases that were wrongly diagnosed as ALL were mostly M0 and M1-AML (Tables 2, 3).

**Table 3:** Cases with non-concordance AML: acute myeloid leukaemia; ALL: acute lymphoid leukaemia; MPO: myeloperoxidase; PAS: periodic acid-Sciff; FCM: flow cytometry

Morphological Diagnosis	No. of Cases	Cytochemistry	FCM Result	FCM Diagnosis
ALL	3	MPO-negative, PAS-few cells show fine granular positivity	CD-34, CD-13, CD33 and CD117	M0-AML with minimum differentiation
ALL	1	MPO-negative, PAS-negative	cMPO, CD-13, CD-34, CD-33 and CD-117	M1-AML without maturation
TOTAL	4			

## Discussion

This study was conducted to diagnose AL in all suspected cases by flow cytometry and to correlate the diagnosis with morphological and special staining (MPO and PAS) techniques. In the present study, all 57 cases of AL were diagnosed with flow cytometry. 87.5% of patients with flow cytometry-diagnosed ALL were diagnosed with ALL and 100% of patients with flow cytometry-diagnosed AML were diagnosed with AML when morphology and cytochemical staining were utilized.

The findings are similar to the previous study by Mhawech et al. [17] who were able to definitively diagnose all 10 of their AML cases using flow cytometry, but only 79.4% of patients were diagnosed with ALL when morphology and cytochemical staining were utilized. The remainder of the cases did not benefit from cytochemistry for diagnosis; hence, flow cytometry was chosen to provide a conclusive diagnosis. Hence, in their study, they stated that "Although cytochemical stains are essential to recognize the subtypes of AML, they are of limited use in differentiating the subtypes of ALL and that the FCM has become a standard tool for the assessment and management of patients with leukaemia."

AL affects all age groups. In this study, the highest incidence was seen in the age group of 0-20 years, constituting 28 cases (49.1%) out of a total of 57 cases. The majority of cases in this age group were ALL (n = 26, 45.6 %). This is in tally with the study by Arora and Arora [18], who found that the median age of children suffering from ALL in India ranged from five to 10 years. Studies by Biondi et al. [19], Reichmann [20], and Arya [21] had similar conclusions. The lowest incidence was seen in more than 70 years, constituting only two cases (3.5%).

AML seems to have a high prevalence as age advances. An observational study by Rajeev et al. [22] found 25.3% of cases above 60 years old. In the current study, there were four cases (13.8%) of AML in the age group above 60 years. There is an obvious male preponderance (35 cases; 61.4%) against 22 cases (38.6%) in the present study. The gender distribution in most of the reported studies by Parekh [23], Bansal et al. [24], Liu et al. [25], and Shoket et al. [26] have a male preponderance, tallying with the present findings. 

It is important to diagnose ALL L3 if the mature B-cells lack CD34 expression. Additional evidence, such as TdT negativity, must also support the diagnosis [26-29]. According to research by Basso et al. [30], immature hematopoietic cells generally express CD34. CD34 is a great marker for tracking blast cell numbers because less than 3% of cells in normal marrow are CD34-positive. B-cell ALL is more common than T-cell ALL in the study population (B-cell ALL: T-cell ALL=1.33:1). The M3-AML (acute promyelocytic leukemia) was the most common. The findings were similar to the findings of studies carried out by Basso et al. [30], Traweek et al. [31], and Paredes-Aguilera et al. [32]. In our study, 72% of cases of AMLs were CD34 positive while 80% of cases of ALL were CD34 positive. According to Traweek et al. [31], leukaemia includes 45 to 65% AMLs and 75% pre-B-cell ALLs that are CD34 positive. Due to its manifestation in lymphoblastic lymphoma and leukaemia of the initial myeloid lineage, it has considerable diagnostic relevance. The neoplastic B-cell fraction in ALL L3 exhibits weak CD34 expression, indicating that these cells have reached maturity and have lost the ability to express CD34 antigen.

In our study 100% of cases of B-ALL were positive for CD19. In their investigation of 74 cases, Paredes-Aguilera et al. [32] evaluated the predictive value of B lineage biomarkers like CD22, CD20, CD19, cyCD22, and cyCD79a. They found that cyCD79a exhibits a sensitivity of 100% and 80% specificity, while cyCD22 showed 90% sensitivity and 88% specificity. They reported that these are very sensitive biomarkers for B-cell ALL patients. Therefore, it is advised to include at least a single of the aforementioned markers in the primary antibody panel for the detection of B-cell ALL. Infants with precursor B-ALL exhibit higher intensity of CD10 expression and less intensive CD20, CD45, and CD34 expression.

The study advantages included complete patient evaluation before study start-up, significant lab protocol optimisation, and great access to advice from our department's flow cytometry experts. Despite the fairly substantial number of markers utilised in this investigation, AL immunophenotyping has employed several additional markers. Parallel cytogenetic analysis would have enhanced the study, but these approaches were not accessible. Additionally, because AML-M6 (erythroleukaemia) and AML-M7 (megakaryoblastic leukaemia) biomarkers weren't included in the study, any instances of these leukaemias may have gone unnoticed.

## Conclusions

In this study, we found that morphological diagnosis by itself is capable of correctly identifying a large proportion of cases of AL, which comprised 92.98% of total cases. There was also a favorable relationship between the findings of flow cytometry and the findings of morphology assessment in determining the distinction between myeloid and lymphoid leukaemias. Only 7.02% of patients required flow cytometry for a precise diagnosis. MPO as well as CD13 were found to be the most frequently expressed AML markers as well. As a result, we can conclude that the potential of flow cytometry to verify morphological diagnoses and more accurately identify lymphoid leukaemias suggests cytochemical stains like MPO and PAS can represent an important additional technique for the medical management of AL in financially constrained institutes. Rapid morphology and flow cytometry interpretation can effectively guide a focused exploration of repeated genetic and molecular abnormalities in AML.
